# P10-08 Effectiveness of social media-based interventions for the promotion of physical activity: scoping review

**DOI:** 10.1093/eurpub/ckac095.147

**Published:** 2022-08-29

**Authors:** Liane Günther, Sarah Schleberger, Claudia Ruth Pischke

**Affiliations:** Institute of Medical Sociology, University of Düsseldorf, Düsseldorf, Germany

**Keywords:** social media, intervention, physical activity, scoping review, behaviour change

## Abstract

**Background:**

A global target of the WHO is to reduce physical inactivity in all adults and adolescents by approximately fifteen percent by 2030. Social media could have an impact in this effort because of its enormous reach, potentially addressing underserved populations in need for PA interventions. To date, characteristics and effectiveness of social media-based interventions for the promotion of PA are not well understood.

This scoping review provides a broad overview of existing social media-based interventions and systematically maps the evidence regarding their effectiveness for PA promotion and other health outcomes.

**Methods:**

Scopus and Medline were searched for the terms ‘physical activity’, ‘social media’ and key social media platforms. Following the PRISMA guidelines for scoping reviews (PRISMA-ScR), two reviewers independently screened abstracts and full texts for eligibility. Any discrepancies were resolved by a third reviewer. All records referring to interventions delivered via any type of social media or at least incorporated one social media component of either pre-existing or study-specific platforms and addressed PA as primary outcome were included. Results were summarized following the objectives of this review. In addition, evidence on use, acceptability, and usability of interventions was mapped.

**Results:**

In total, 12.321 publications were identified and 53 met the inclusion criteria. The use of Facebook was most prevalent in interventions to promote PA, followed by the incorporation of study-specific platforms alone or in combination with Facebook. Occasionally utilized applications included Twitter, Pokémon Go, and YouTube. More than one third of the studies revealed positive effects regarding the promotion of PA. Additional evidence emerged in this scoping review demonstrating that social media-based interventions have a positive impact on other physical dimensions of health (e.g. weight or blood pressure). Due to the great heterogeneity in the assessment of acceptability, use, and usability across the different publications, results cannot be pooled. However, the use of Facebook as a motivator for PA was rated positively across six of the included studies.

**Conclusions:**

Social media seems to be a promising tool for increasing PA at the population level. Future studies should take the abundance of platforms into account and select social media interventions meeting the requirements, preferences, and needs of varying target populations.

